# A user-friendly workflow for analysis of Illumina gene expression bead array data available at the arrayanalysis.org portal

**DOI:** 10.1186/s12864-015-1689-8

**Published:** 2015-06-30

**Authors:** Lars M.T. Eijssen, Varshna S. Goelela, Thomas Kelder, Michiel E. Adriaens, Chris T. Evelo, Marijana Radonjic

**Affiliations:** Department of Bioinformatics-BiGCaT, Maastricht University, Universiteitssingel 50, 6229 ER Maastricht, The Netherlands; TNO, Research Group Microbiology & Systems Biology, Utrechtseweg 48, 3704 HE Zeist, The Netherlands; EdgeLeap B.V., Hooghiemstraplein 15, 3514 AX Utrecht, The Netherlands; Department of Experimental Cardiology, Academic Medical Center, Meibergdreef 9, 1100 DD Amsterdam, The Netherlands; Current address: Charles River Laboratories, BioFocus, Discovery Services, Darwinweg 24, 2333 CR Leiden, The Netherlands

**Keywords:** Microarray, Illumina bead array, Transcriptomics, Data analysis, Normalization, Quality control

## Abstract

**Background:**

Illumina whole-genome expression bead arrays are a widely used platform for transcriptomics. Most of the tools available for the analysis of the resulting data are not easily applicable by less experienced users. ArrayAnalysis.org provides researchers with an easy-to-use and comprehensive interface to the functionality of R and Bioconductor packages for microarray data analysis. As a modular open source project, it allows developers to contribute modules that provide support for additional types of data or extend workflows.

**Results:**

To enable data analysis of Illumina bead arrays for a broad user community, we have developed a module for ArrayAnalysis.org that provides a free and user-friendly web interface for quality control and pre-processing for these arrays. This module can be used together with existing modules for statistical and pathway analysis to provide a full workflow for Illumina gene expression data analysis.

The module accepts data exported from Illumina’s GenomeStudio, and provides the user with quality control plots and normalized data. The outputs are directly linked to the existing statistics module of ArrayAnalysis.org, but can also be downloaded for further downstream analysis in third-party tools.

**Conclusions:**

The Illumina bead arrays analysis module is available at http://www.arrayanalysis.org. A user guide, a tutorial demonstrating the analysis of an example dataset, and R scripts are available. The module can be used as a starting point for statistical evaluation and pathway analysis provided on the website or to generate processed input data for a broad range of applications in life sciences research.

**Electronic supplementary material:**

The online version of this article (doi:10.1186/s12864-015-1689-8) contains supplementary material, which is available to authorized users.

## Background

Illumina bead arrays [1] are a popular choice for array-based genome profiling studies. Although Next Generation Sequencing technology is on the rise, microarray-based gene expression profiling is still widely utilized due to its ease of use, robust performance, reproducibility, and low per-sample cost. Furthermore, open data repositories (e.g. ArrayExpress [2] and Gene Expression Omnibus [3]) contain a vast amount of microarray experiments, which are often re-analyzed, integrated, or combined with newly generated data in the context of modern integrated systems biology research. This process is facilitated by easy access to streamlined processing. To extract biologically meaningful information from genome profiling experiments, generated data first needs to be quality checked, filtered, pre-processed and statistically analyzed. Having these basic analysis steps at a user’s disposal is essential for an effective and iterative research process. As gene expression profiling experiments are typically designed, performed, and interpreted by biological domain experts rather than bioinformaticians, it is important to enable these researchers to independently operate basic analysis pipelines. Pipelines with a user interface that provides immediate and intuitive feedback are of great interest for increasing efficiency and effectiveness of the research process. Besides proprietary vendor-provided software (BeadStudio, GenomeStudio) and open-source software *Illuminaio* [4], several pre-processing and quality control (QC) methods for Illumina bead arrays are available (*beadarray* [5]; *lumi* [6]; *limma* [7]). However, utilization of these methods requires extensive bioinformatics skills and therefore they are not readily accessible for a broad researchers community. To extend utility of analysis workflows for Illumina bead arrays also to non-bioinformaticians, we have created an open-source, user-friendly workflow, accessible via the web interface of ArrayAnalysis.org, that combines functionality of Bioconductor packages for essential quality control and pre-processing, with statistical functions and downstream analysis [8].

The relevance of analysis workflows for Illumina bead arrays that are friendly to a wide range of researchers has been recognized by several other bioinformatics developers, resulting in availability of tools and pipelines related to our work (e.g. Chipster [9], MadMax [10], IlluminaGUI [11]). Nevertheless, our module for ArrayAnalysis.org provides a significant contribution to the research community as it provides an easily accessible alternative that does not require local installs. For instance, Chipster provides similar functionality but requires local software installation and availability of specific Java versions; Madmax is not open source and requires login credentials to be provided by the developers; and IlluminaGUI requires a local install of R and its support has been discontinued. Therefore, our web interface-based workflow is a convenient resource for free, fast and user-friendly analysis of Illumina bead arrays by a broad community of researches - regardless of their bioinformatics skill level or research budget.

## Implementation

**Fig. 1:**
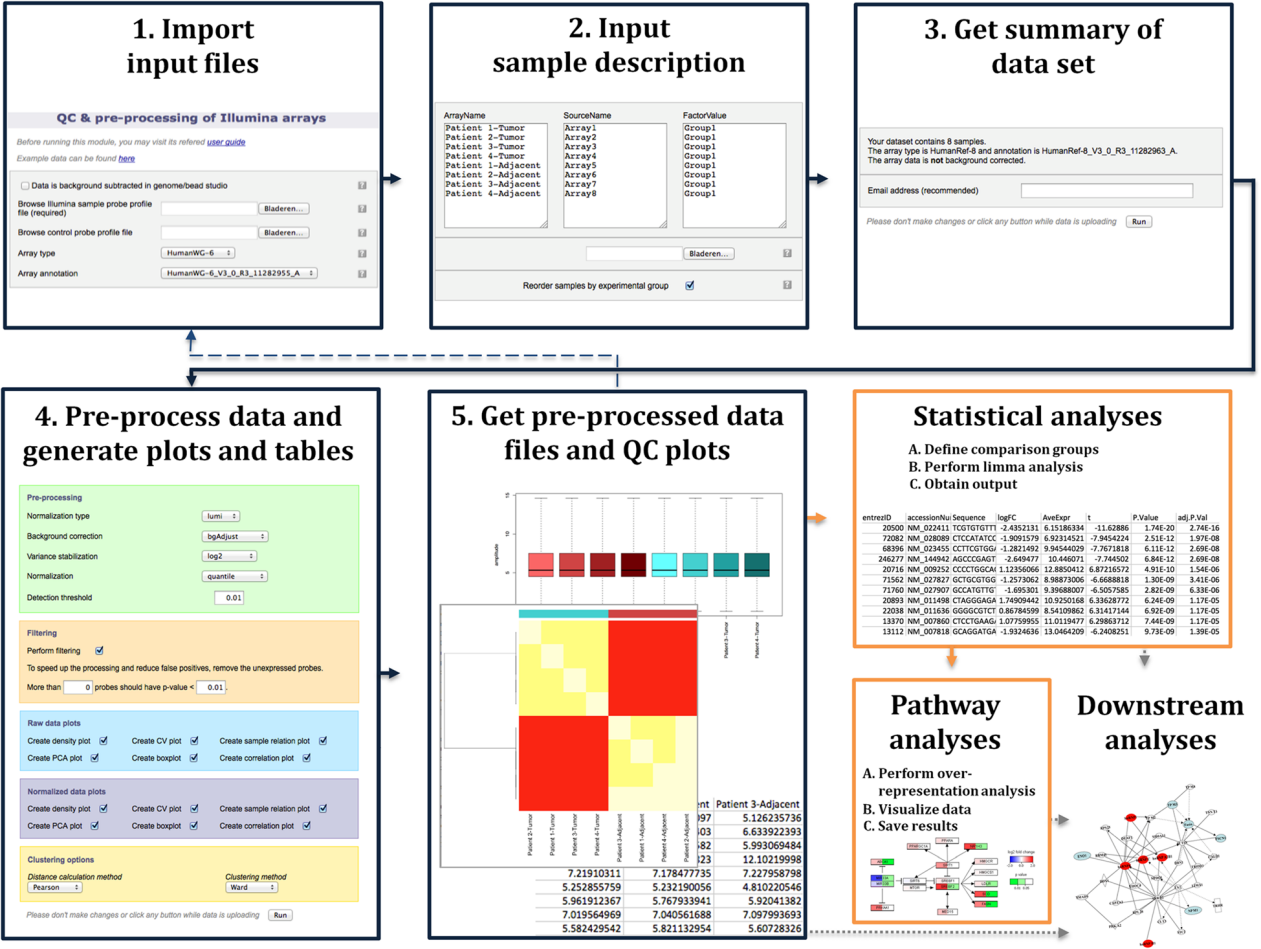
Schematic representation of the different steps in the data workflow for Illumina bead arrays. Steps 1–5 are part of the newly added Illumina QC and pre-processing module. (1) Import the input data text files; (2) Enter sample annotation and experimental grouping, by either uploading a description file or completing the form (array names are pre-filled based on the data set uploaded); (3) Summary of the array type, species and number of arrays found in the data set and whether data has been background corrected, with an option to enter an email address for notification when the workflow has finished; (4) Select a pre-processing method and the plots and tables to be computed on the raw and pre-processed data; (5) Obtain the normalized data table and diagnostic images to check the data quality. Illustrative examples of such tables and images are shown (array boxplots, array correlation heatmap, head of normalized data table). Optionally, steps 1 to 5 of the pre-processing procedure may be repeated after exclusion of low quality arrays. The output files can be further used to perform statistical analyses through the statistics module and pathway analysis through the pathway analysis module, or to proceed with downstream analyses in external tools

The Illumina QC and pre-processing module was developed to complement and link to previously created modules for analysis of microarrays, available at www.arrayanalysis.org [8]. The Illumina module has been implemented as a wizard guiding the users through the different steps and is connected in an ArrayAnalysis workflow to downstream modules for statistics and pathway analysis. Figure 1 shows an overview of the steps of the Illumina module and its use together with other modules and software.

The module was implemented using R and Bioconductor packages for Illumina analysis *lumi* [6] and *limma* [7] to provide the user with the most commonly used analysis options. Using the *lumi* package, we implemented various types of background correction (e.g. ‘none’, ‘bgAdjust’, ‘forcePositive’), variance stabilization (‘vst’ (variance-stabilizing transformation), ‘log2’, ‘cubicRoot’) and normalization. Additionally, the *neqc* method from the *limma* package has been included, which performs a background correction using a normal-exponential-modeling approach [12] followed by a quantile normalization of all regular and control probes together, and a log2-transformation on the dataset. After normalization, probes with intensities below detection level can be removed to speed up the processing and to reduce false positives.

Five types of quality control (QC) plots are implemented: (1) density plots and (2) boxplots of the log-intensity distributions of all arrays on a single graph, facilitating comparison of signals between arrays and identification of arrays with deviating distributions; (3) a correlation coefficient plot, representing correlations between all pairs of arrays in the dataset as a colored matrix; (4) a principal component analysis (PCA) plot, providing another view of the correlations of expression between arrays: the data are projected on several axes (or components) that explain the largest amounts of variance; (5) a hierarchical clustering plot that can be generated using various distance metrics (Pearson, Spearman, or Euclidean) and clustering methods (Ward, Mcquitty, average, median, single, complete, or centroid), and is used to inspect the groupings of the samples. All plots use consistent colors for arrays and experimental groups and can be generated for both raw and pre-processed data, which helps to assess whether the pre-processing step corrects possible aberrations.

The Illumina identifiers are converted to equivalent nucleotide universal identifiers (nuIDs) [13] based on their probe sequence. After quality control and pre-processing, the nuIDs are used to add additional annotation (e.g. gene symbol, Entrez Gene identifier, etc.) to the processed result tables.

## Results and discussion

When running the Illumina workflow, the user is guided through the different analysis steps via a web based user interface. At the first step, the user is prompted to upload a summarized probe-level data file and optionally a control probe data file, the output of Illumina’s BeadStudio/GenomeStudio software. The user may choose to perform all pre-processing steps within our workflow (recommended), or to provide already background-subtracted data. Both summarized probe-level and summarized gene-level input data are supported. Summarized probe-level data is recommended as input, as this will eliminate the occurrence of improper combinations of the expression values of different probes into a single-gene value [14].

In the second step, the user can annotate the imported samples by entering custom sample names and experimental group names by either uploading a sample description file or entering the sample description information manually via the web based interface.

The third step summarizes the information about the uploaded data and provides the user with the option to enter an email address for notification when the workflow has finished.

The fourth step will perform background correction and normalization of the user’s data. This encompasses the removal of per-array technical effects, which ensures that the values being further analyzed reflect underlying biology. Three actions are typically performed to achieve the following [14]: (i) background correction, (ii) between-array normalization and (iii) data transformation (typically a log2-transformation). The user may choose between two popular pre-processing approaches that implement these actions for Illumina data: (a) *lumiExpresso* from the *lumi* Bioconductor package [6], or (b) *neqc*, from the *limma* package [7]. Also, the user can choose the types of plots that are to be created and whether filtering probes with intensities below detection level is to be performed.

Upon completion of the run, the user receives a link to download a zip archive of results either at the web-interface or by email. If the QC diagnostic plots show arrays of insufficient quality, the pre-processing procedure may be repeated after exclusion of those arrays. Otherwise, the user can immediately proceed with the next module of the workflow to perform statistical analysis. Via a web interface, the existing statistics module prompts the user to specify which experimental groups are to be compared (e.g. treated versus control) or to define any custom comparison of interest. After submitting the choices, this module runs *limma* model fitting to compute a table of relevant statistics, including estimated coefficients (effect sizes) and their significances [7]. Results from the statistics module can then be used for further pathway analysis processing in a downstream module that makes automated calls to PathVisio [15] or they can be downloaded for processing in other software.

Running time of an analysis is very much dependent on the size of the input file, the number of arrays, the specific user settings, and the modules used, and will range from minutes to hours in the extremes. Performance of ArrayAnalysis servers is being monitored to make sure they effectively deal with the workload, and extra capacity can be allocated in future if needed. When not surpassing a dozen concurrent runs, running times will not increase much. Additionally, users can download the R scripts to run on their own systems if desired, for example in case of many projected runs or very large data sets that would not be convenient to process over the internet. The scripts have been designed for ease-of-use, providing a separate initiation script to specify user settings (e.g. data directories and preferences), which automatically calls the other scripts.

The addition of the currently introduced Illumina module complements ArrayAnalysis.org with functionality to pre-process data from experiments run on the widely used Illumina bead array platform. It provides users of this platform or those processing existing data not only with an easy to use data quality control and pre-processing web module, but also with a direct connection to further modules offering downstream statistical and pathway analysis functionalities. As a whole, ArrayAnalysis.org is continuously being improved, evolving into a one-step solution for pre-processing, statistical analysis, and biological interpretation of data from multiple technological platforms. Being an open source project, developers within the user community can contribute by adding modules or improving functionality of existing ones, and source code can be downloaded for local deployment.

## Conclusions

The developed Illumina bead array analysis workflow provides an easy, fast, and intuitive way for quality control, pre-processing, statistical, and pathway analysis of Illumina gene expression arrays for a broad range of researchers. The workflow provides immediate feedback on quality and basic statistics outcomes of generated data, increasing the speed and iterative capacity of intuitive research pipelines. This enables researchers to effectively resolve the first steps in data analysis and focus on their primary interest: extracting biologically meaningful information out of their gene expression data. The workflow can therefore be used as a starting point facilitating a broad range of applications in life sciences research.

## Availability and requirements

- Project name: ArrayAnalysis.org Illumina Pre-processing and QC module
- Project home page: http://www.arrayanalysis.org
- Operating system(s): Platform independent (web-based)
- Programming language: implemented in R, php
- Other requirements: none
- License: Apache version 2.0
- Any restrictions to use by non-academics: no restrictions
- User guide: Additional file 1.
- Tutorial: Additional file 2.
- Source code: https://github.com/BiGCAT-UM/ilmnQC_Module (most recent version) and Additional file 3.

## Additional files

The Additional files are given for reference, most recent versions are available from http://www.arrayanalysis.org and https://github.com/BiGCAT-UM/ilmnQC_Module. Additional file 1:
**User guide.**
Additional file 2:
**Tutorial demonstrating analysis of a publicly available example dataset from ArrayExpress.**
Additional file 3:
**R scripts.**

## Abbreviations

QC: Quality control
Limma: Linear models for microarray data
PCA: Principal component analysis
nuID: Nucleotide universal identifier
