# Supplementary material for: A user-friendly workflow for analysis of Illumina gene expression bead array data available at the arrayanalysis.org portal
Source: BMC Genomics. 2015 Jun 30;16(1):482. doi: 10.1186/s12864-015-1689-8 (PMC4486126; doi:10.1186/s12864-015-1689-8)

USER GUIDE

ARRAYANALYSIS.ORG – ILLUMINA PRE-PROCESSING PIPELINE - DOCUMENT VERSION: 1.0.0

Table of Contents

Overview of the documentation 2
Bug tracking system 2
Example dataset......2
More information 2
Input files 3
Use of the on-line Illumina pre-processing pipeline......3
First step:......3
Second step: describe the dataset......4
Third step: define your analysis......5
Execution step 7
Results......7
Graphs description 8
Boxplots of log-intensities......8
Density histogram of log-intensities......9
PCA analysis plot......10
Hierarchical clustering plot......12
Correlation plot 13

Overview of the documentation

This guide will help you in the installation and/or use of the QC & pre-processing of Illumina arrays pipeline.

All source code has been written in R and is open-source, available under the Apache License version 2.0. It is available on our Download page.

Illumina pre-processing pipeline can be run:

- on-line via the arrayanalysis.org webportal (follow "Get started") or
- locally as an automated R workflow consisting on a R function.

The main functions of the Illumina pre-processing pipeline are:

- To compute array quality information;
- To plot images that allow identifying any aberrations present in the dataset;
- To return pre-processed data and QC plots.

Bug tracking system

If you encounter an issue by using the code, you can report it at any moment using the Google docs link below to report your problem (with a screenshot if possible):
https://docs.google.com/document/d/13SOGO9svBPUfG36nPAIvaC-GkelZttk0VV50eto4cMc/edit

Example dataset

A not background subtracted example dataset is available on the website in zip format. Extract the files and use them as input for the pipeline.

Description of the example dataset:

Tumor-adjacent Dataset (HumanRef-8 V2) - This dataset contains samples from four patients, each with a matched tumor and adjacent sample (8 samples total). These samples were labeled with the Ambion TotalPrep kit and hybridized to the HumanRef-8 v2 array.

More information

The paper mentioned below contains more information about the Illumina bead array platform and analysis of the data in different ways. It will also tell you the current view concerning the best way to analyze Illumina BeadArray data using the Bioconducter project.

BeadArray expression analysis using bioconductor.
Ritchie ME, Dunning MJ, Smith ML, Shi W, Lynch AG.
http://www.ncbi.nlm.nih.gov/pubmed/22144879

Input files

The files needed for this pipeline must be extracted from the Bead/Genome Studio software. An example of the input data format is available in the zip file on the website.

Data are exported from this application in tab-delimited files (separate files for the experimental and control probes) with each row giving the summary information for a particular probe, and different columns for each sample. We recommend exporting raw summary values (which have not been background corrected, transformed, or normalized) at the probe level ("probe profiles"). The pipeline can also be used to pre-process data which has been background subtracted.

The columns **essential** for the pipeline are the columns containing the AVG_Signal and BEAD_STDEV. We also recommend choosing the extraction of the PROBE_SEQUENCE information. The control file can be extracted using the standard columns shown in the Bead/Genome Studio Software Before uploading the file(s) in the Illumina pipeline check if these columns are present in your input file.

Use of the on-line Illumina pre-processing pipeline

You can access the on-line pipeline on arrayanalysis.org webportal: follow "Get started" . **JavaScript** has to be enabled (activated) in your web browser. You will be warned if it is not the case. You can activate it at any time in the browser options (see activatejavascript.org if needed)

The on-line pipeline contains three steps before the launch of the analysis:

- First step: Is your data background subtracted: load a sample probe profile text file if your file is not background subtracted the pipeline will also ask for a control probe profile text file
- Step 2 (4): Then you complete the description of the dataset
- Step 3 (5): And finally you choose the plots to be computed and their parameters.

Then:

- Execution (7): The pipeline is executed with the settings you choose
- Results: You get the results after the execution step in a zip, or by e-mail.

The interrogation mark button will help you by giving you a contextual help. Note that this feature is available when JavaScript is activated and is not yet supported by Google Chrome and Safari browsers.

First step:

The following picture shows the screen for the first step:

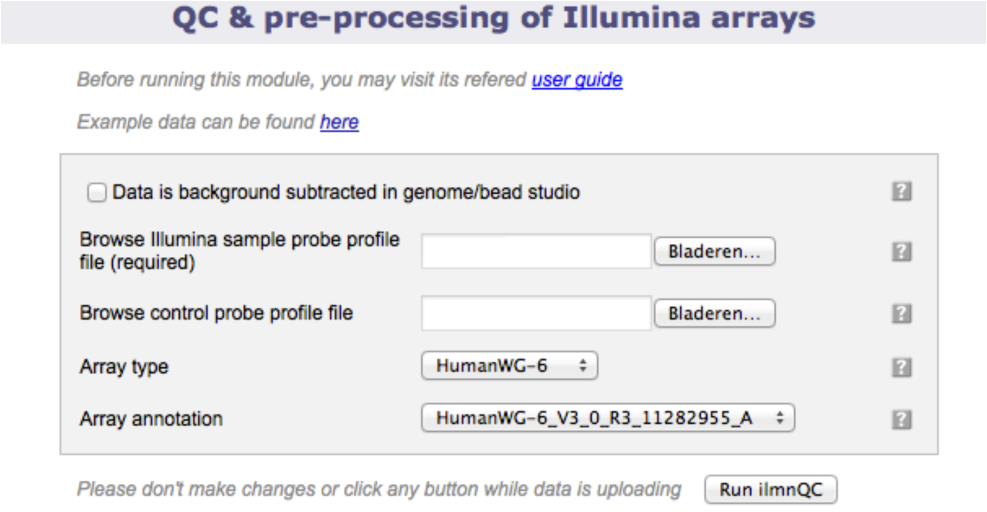

Check the box above this form if you have a background subtracted sample probe profile text file. Browse to your data files for upload of the files to the server.

Select the appropriate array and array annotation type and click on “Run ilmnQC”.

When the file is loaded without error, you are automatically directed to the next step. Otherwise you get a message indicating the error encountered. (This check for file upload is not available)

Second step: describe the dataset

The following picture shows the screen obtained after completing the first step:

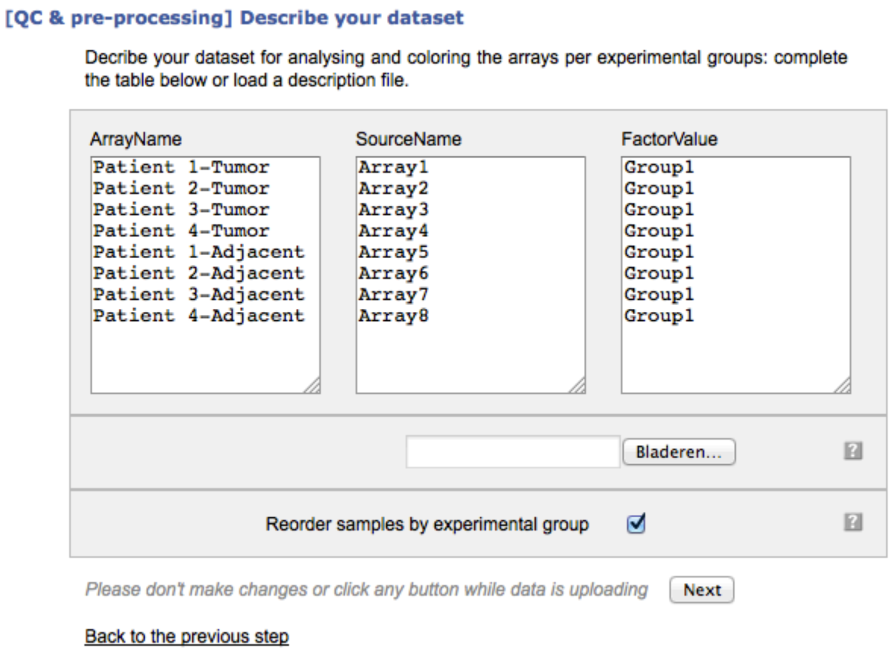

Your dataset has been read and the following information is presented in a three columns table:

- Column "ArrayDataFile" contains the array file names of your N arrays found in the input uploaded text file. You **cannot** edit this column.
- Column "SourceName" is filled with Array1 ... ArrayN. These names will be used for the analyses. Feel free to modify these names at the condition you can only use **unique** names.
- Column "FactorValue" is always set to "Group1". If you want your array groups to be represented in the analyses and plots, rename the factor groups.

You may also prefer to enter directly this information from a file you have prepared. If this is the case, browse your description file (saved in tab-delimited text format) in the second section. If you enter such a file the information contained in the previous table will be skipped. The columns needed are:

1) The actual names or identifiers of the arrays. This list must be unique.
2) The names or identifiers that you want the arrays to have. This list is also used for the creation of the plot labels; also this list must be unique.
3) The last column is used to group your arrays. This list does not have to be unique.

The description file must always have the following format:

ArrayDataFile	SourceName	FactorValue
6668263018_A	Patient 1-Tumor	Tumor
6668263018_B	Patient 2-Tumor	Tumor
6668263018_C	Patient 3-Tumor	Tumor
6668263018_E	Patient 4-Tumor	Tumor
6668263018_F	Patient 1-Adjacent	Adjacent
6668263018_G	Patient 2-Adjacent	Adjacent
6668263018_H	Patient 3-Adjacent	Adjacent
6668263026_A	Patient 4-Adjacent	Adjacent

The last section of step 2 proposes you to reorder the arrays per groups, which is done by default. Thus all the arrays representing the same factor will be grouped together on the plots. If you uncheck the checkbox, arrays will be ordered as they were in the raw data file.

Clicking on the "Next" button will direct to the last step if no error has been detected.

Third step: define your analysis

The contextual help is not any more given by the interrogation mark buttons: help messages will pop up as soon as you activate a field (for example if you click in a text field or check a checkbox)

This last input form is divided into three main sections: the first part allows a quick launch, the second part defines in details the pre-processing parameters applied to the raw data (normalization, filtering and re-annotation parameters) and the last part is dedicated for the creation of the QC graphs.

First part of the input form

The following picture presents the first part; it recalls briefly what your dataset contains and asks you to enter an e-mail address.

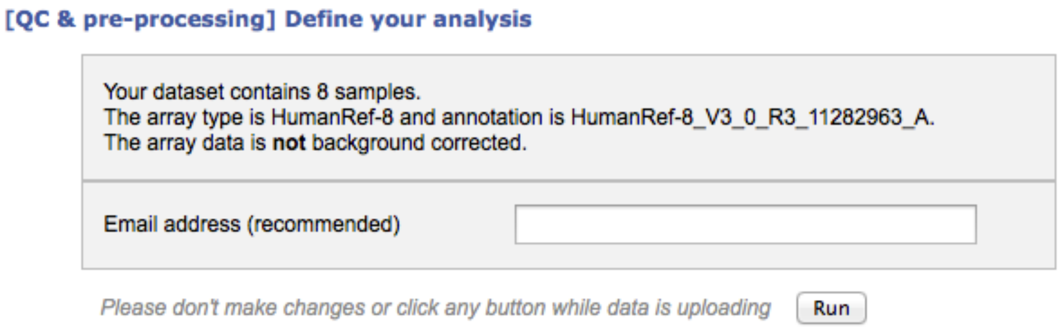

This is optional: if you don't enter your e-mail, you will need to keep the browser opened and not close the page before the end of the calculation. On the contrary, if you enter your e-mail address - which is recommended - you can close the windows as soon as the next page appears and you will be inform of the end of the analysis by e-mail. You would just have to follow the links to the result files given in the e-mail.

You may launch the analysis with the "Run" button right after this first section. In this case default parameters will be used.

Second part of the input form

This part contains three frames representing the three analysis applied to your raw data:

1. **Pre-processing:**
   You can choose to do this using the *lumi* of the *limma::neqc* function.

   The neqc function performs normexp background correction and quantile normalization aided by control probes.

   Choosing the lumi function will give you the option to select the type of background correction, normalization and variance stabilization. The lumi function has the following normalization options:

   The method 'bgAdjust' is designed to approximate what BeadStudio does for background adjustment. In the case when 'log2' transform is used in the background correction method ('forcePositive') will be automatically used, which basically adds an offset (minus minimum value plus one) if there is any negative values to force all expression values to be positive.

2. **Filtering of the pre-processed data file:**
   To speed up the processing and reduce false positives, you can remove the unexpressed probes. The least stringent values are already pre-filled. Beads detection pvalue must be smaller then 0.01) and at > 0 (detect probes) samples should have this p-value.

3. **Creation of a annotation file**
   Will create an annotation file for the normalized data file.

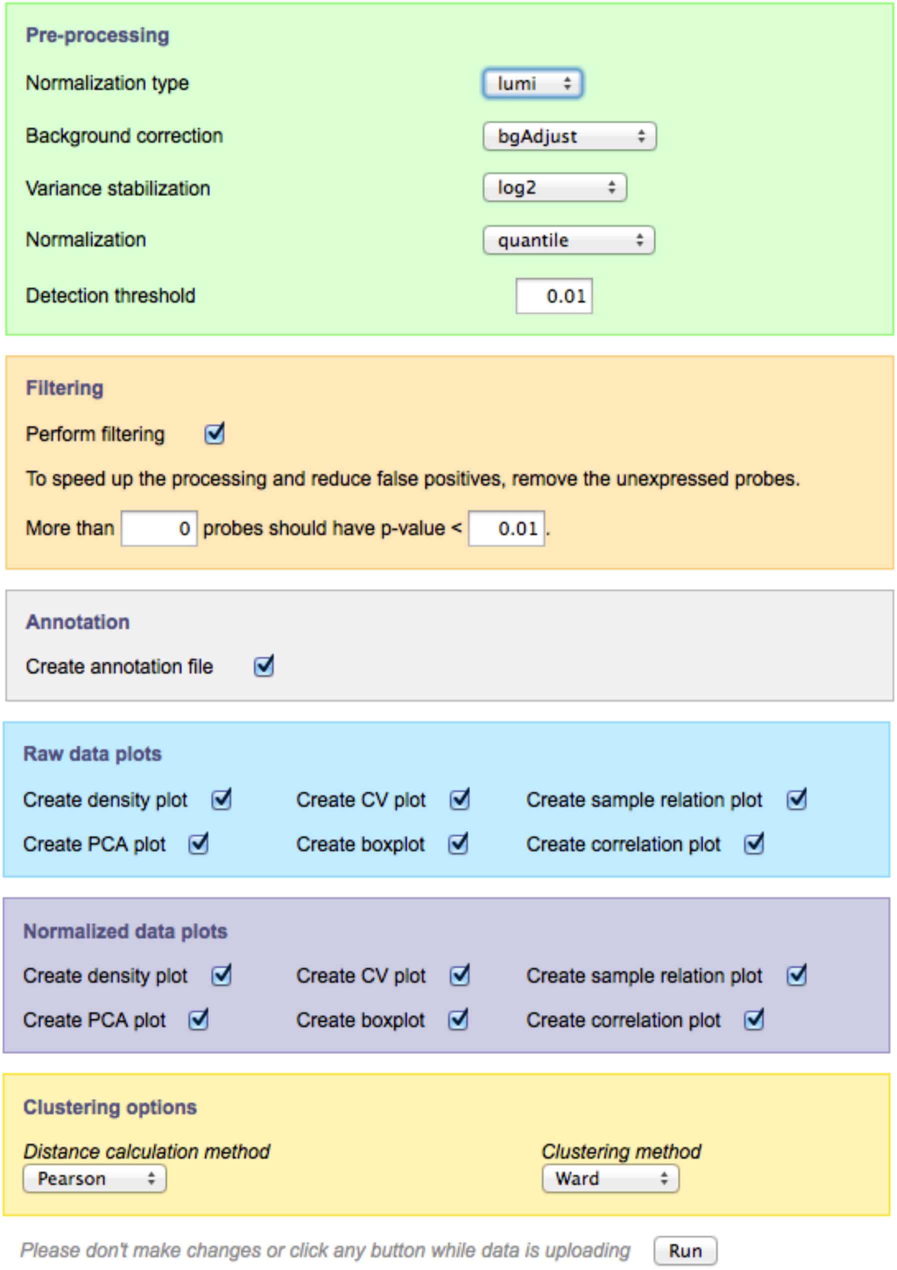

Last part of the input form

Most of the parameters are checkboxes that you would check or uncheck to indicate whether a certain plot or table has to be computed or not. The analyses and plots are described in the last paragraph of this guide. Some analyses or plot construction, such as the hierarchical clustering, need particular parameters. You may modify the default values in the last section of the form.

Once the input form is completely filled, you can launch the analysis with the "Run" button. Don't click any button after clicking on the "Run" button and before being automatically redirected to the execution page, otherwise you may compromise your analysis.

Execution step

After the third step, the Illumina pre-processing pipeline has all it needs to launch the analysis. The page become grey with a message telling you that the analysis is running. If you entered your e-mail address in the previous step, you can close the window.

You will find on this page a recalling of the choices you made for this analysis: which files were loaded or created, which plots you decided to create for raw and normalized data and how you managed the pre-processing step (not all the options from the 3th input form are presented).

The following picture shows the screen for the execution step:

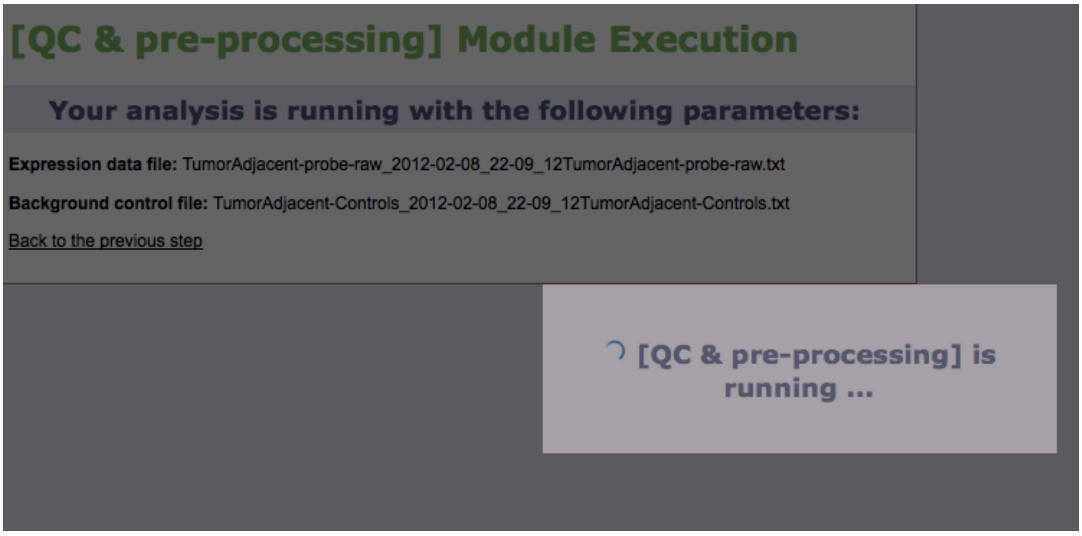

Results

If you keep your browser open the browser will update into a result download page. It will show a link to download all the files in a zip format and it will show a recall of all the parameters used by R. If you filled in you're email address you can download the result zip file by the link in the email you have received.

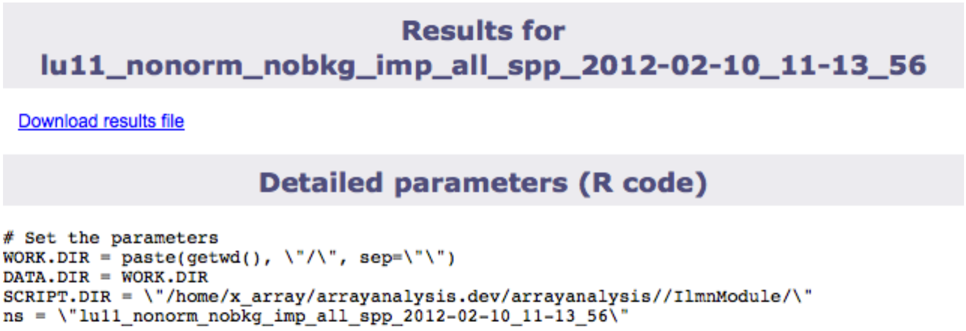

Graphs description

Boxplots of log-intensities

Boxplots of log-intensity distribution are plotted for between-array comparison.

Figure 1 box plot of raw intensities

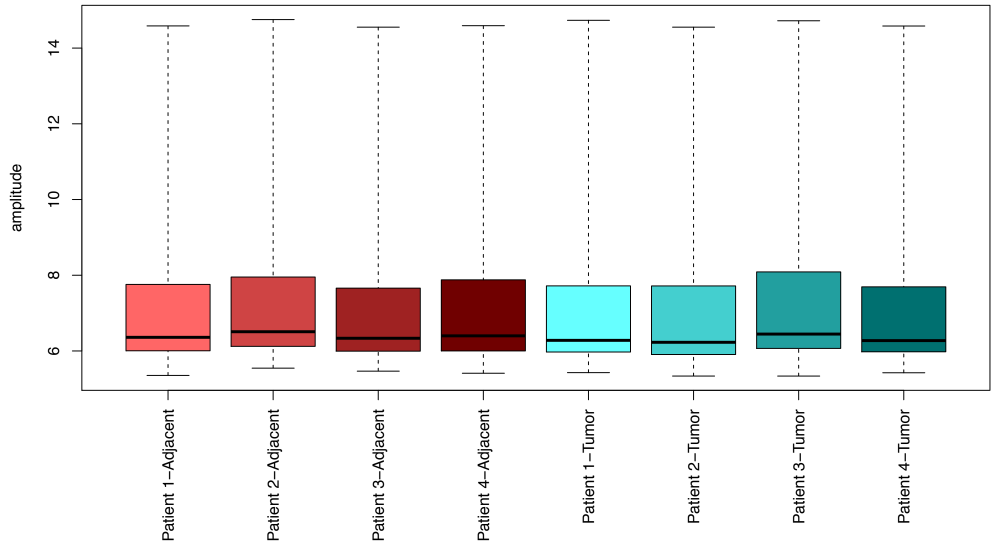

Figure 2 boxplot of normalized intensities

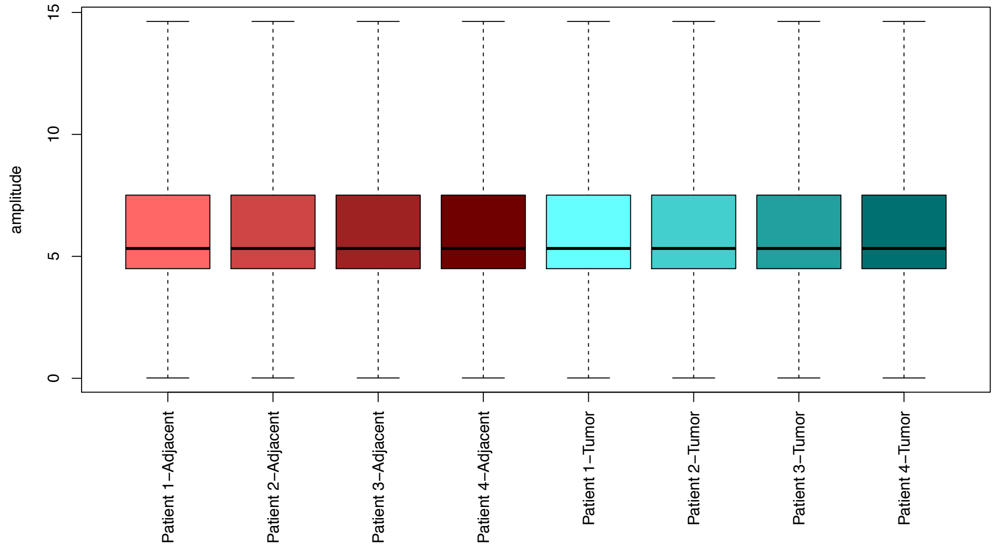

Density histogram of log-intensities

Density plots of log-intensity distribution of each array are superposed on a single graph for a better comparison between arrays and for an identification of arrays with weird distribution. As for the boxplots, the density distributions of raw log-intensities are not expected to be identical but still not totally different while the distributions of normalized probe-set log-intensities are expected to be more identical. Drawing these plots before and after normalization allows also checking the normalization step. The pipeline will also create a density plot of coefficient of variance for both raw and normalized data.

Figure 3 Density plot of raw values

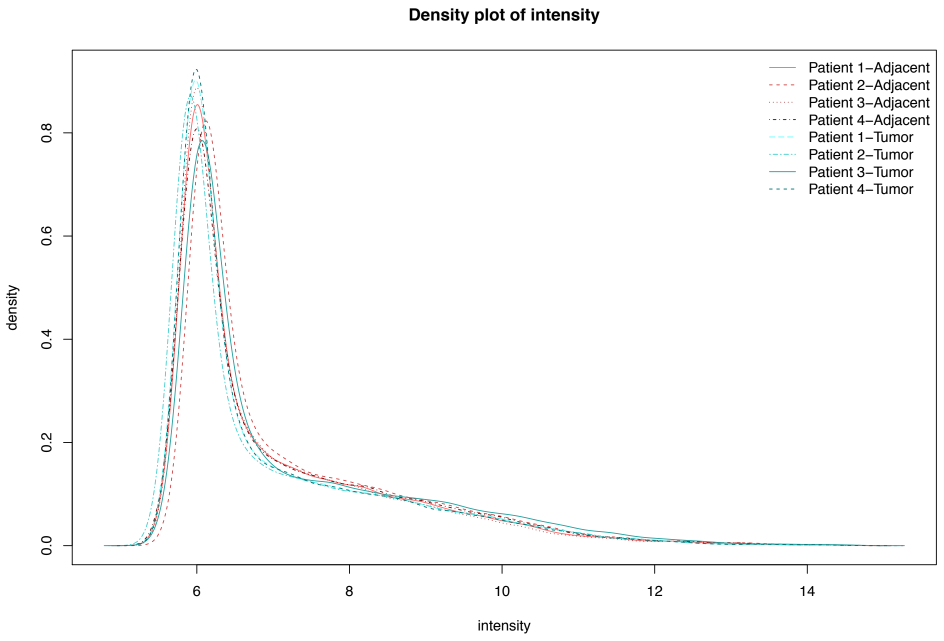

Figure 4 Density plot of normalized values

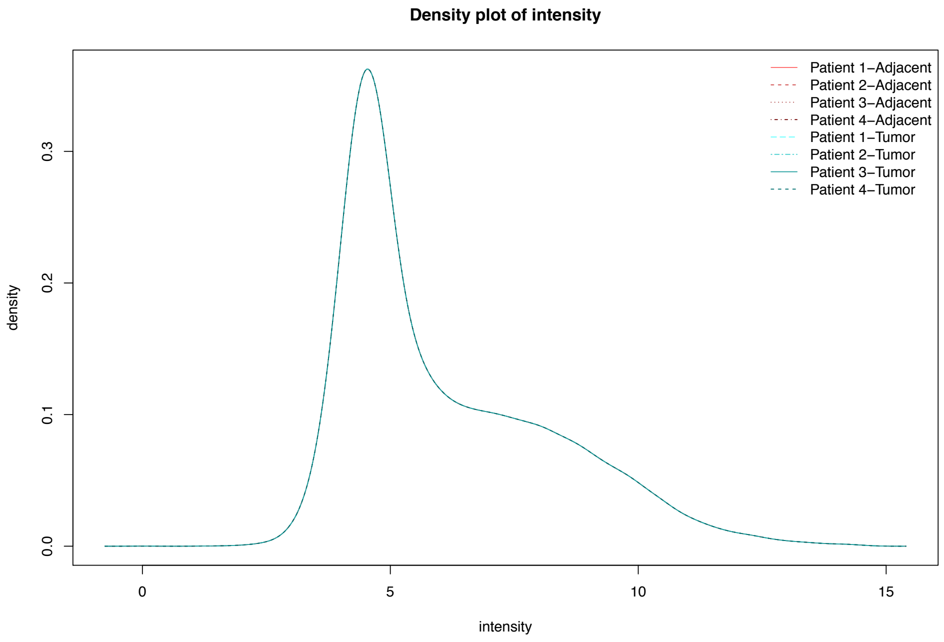

PCA analysis plot

The PCA (Principal Component Analysis) gives another view of the correlations of expression between arrays: the data are projected on several axes (or components), ordered by decreasing significativity; the first principal component (PC1) explains most of the variations of expression. Clusters of samples on a PCA plot present a strong correlation of expression signals. This analysis is proposed before and after normalization.

Figure 5 PCA analysis of raw data

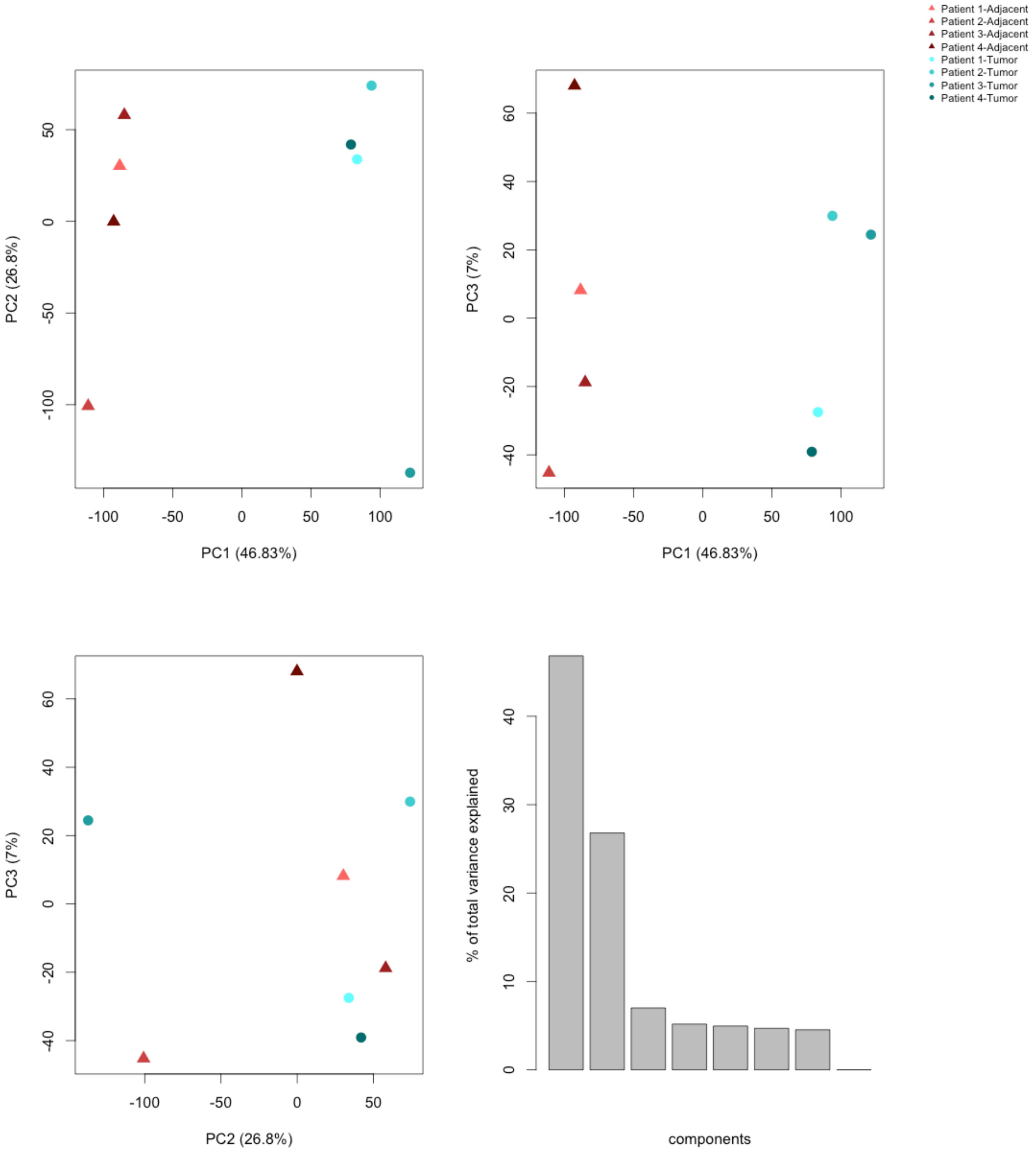

Figure 6 PCA analysis of normalized data

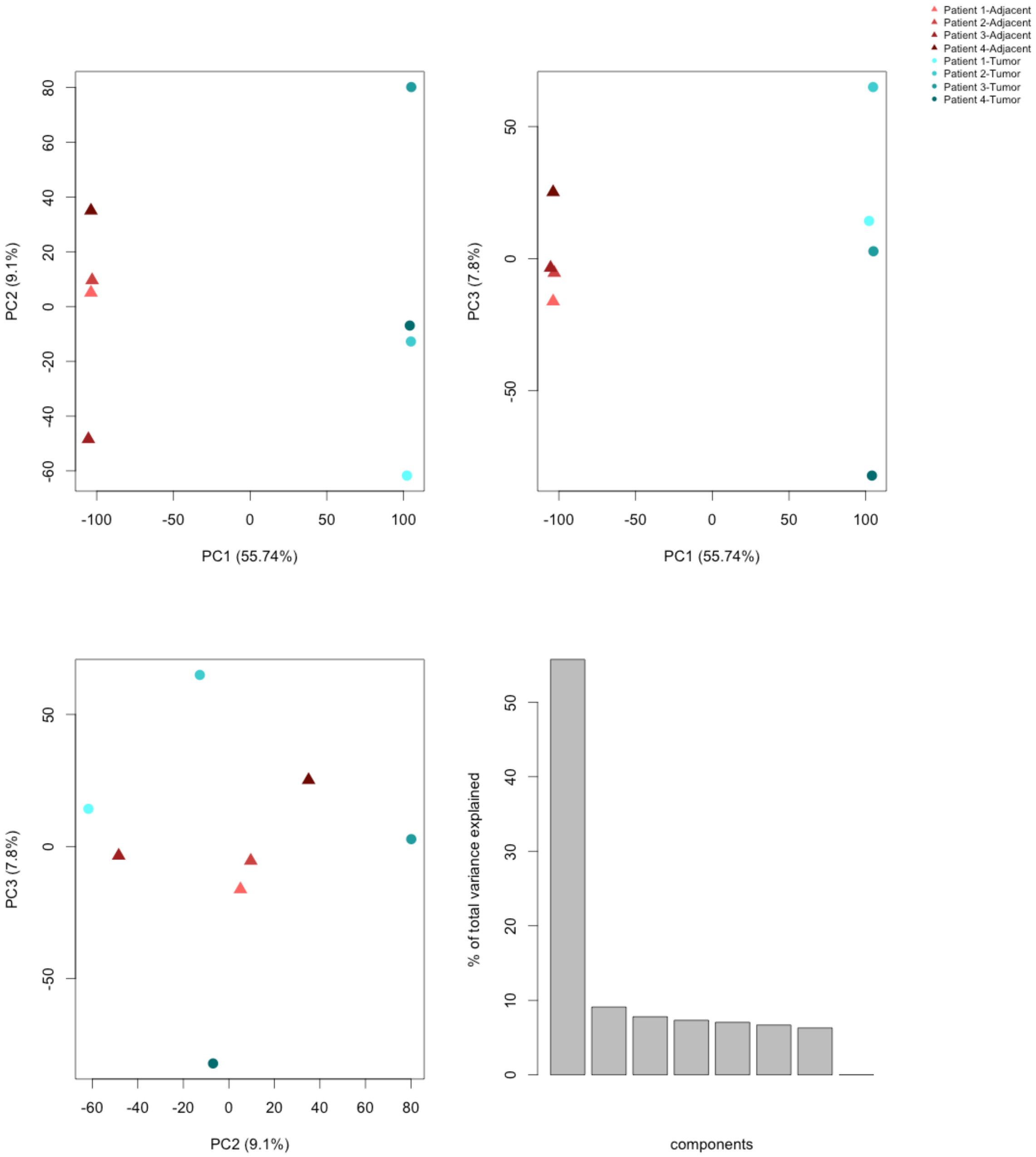

Hierarchical clustering plot

The Hierarchical Clustering plot is computed in two steps: first it computes an expression measure distance between all pairs of arrays and then it creates the tree from these distances. The distance absolute values are of interest as well as the groups of arrays that emerged from this analysis.

Figure 7 Clustering of raw data intensities

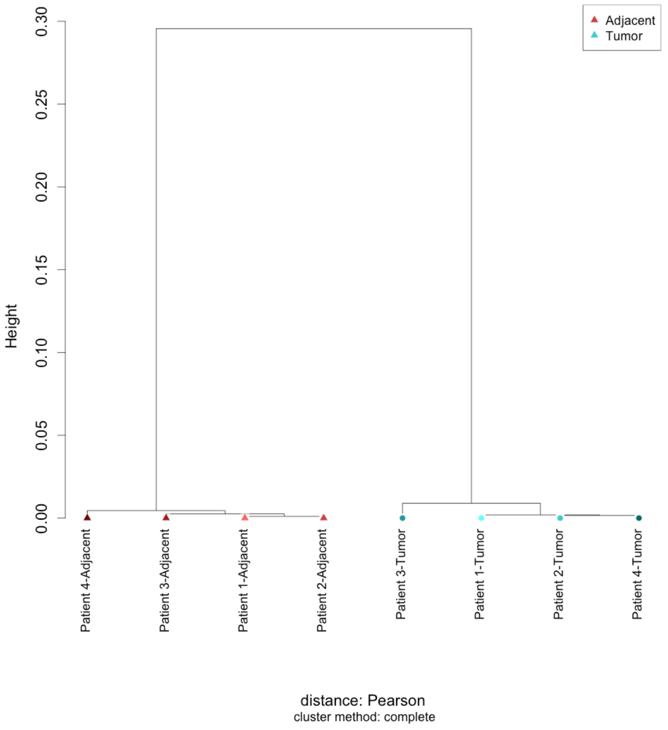

Figure 8 Clustering of normalized data intensities

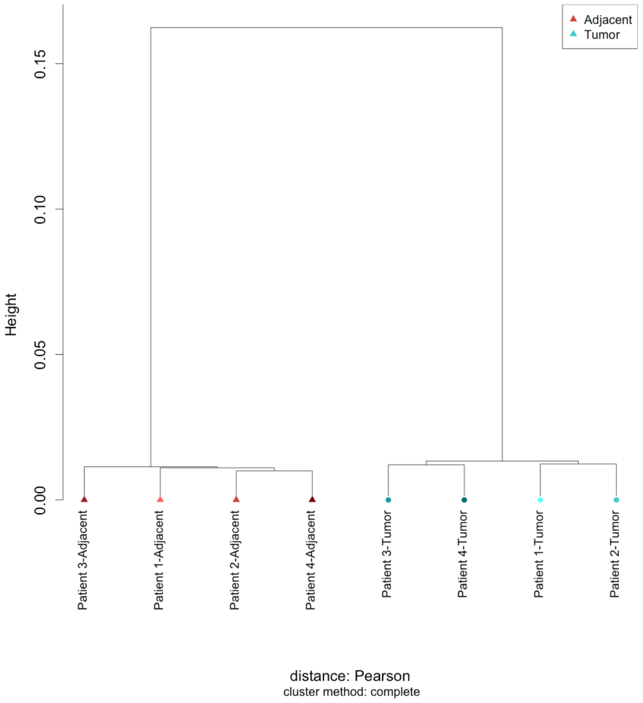

Correlation plot

A correlation coefficient is computed for each pair of arrays in the dataset and is presented qualitatively on a colored matrix. The minimal value of this coefficient (given on the legend) gives a good idea of the dataset homogeneity: low coefficients indicate important differences between array intensities. We suggest plotting correlation before and after normalization: as the normalization makes the arrays more comparable, the correlation should be higher after this step.

Figure 9 Correlation plots for raw data

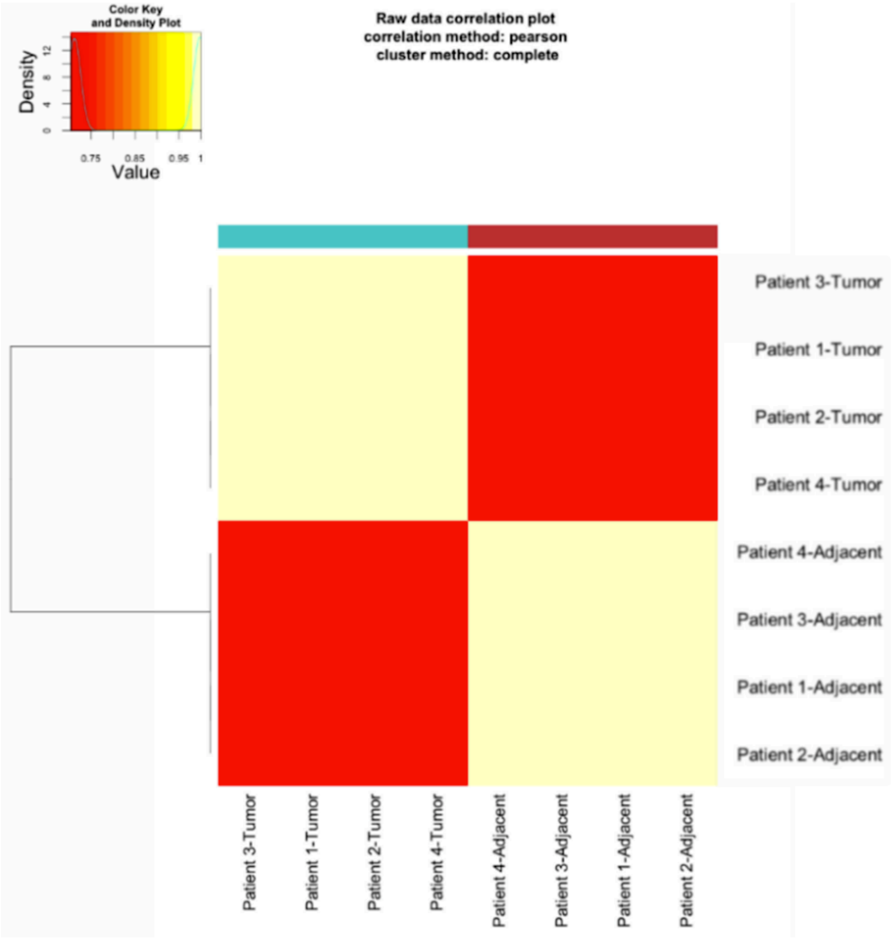

Figure 10 Correlation plots for normalized data

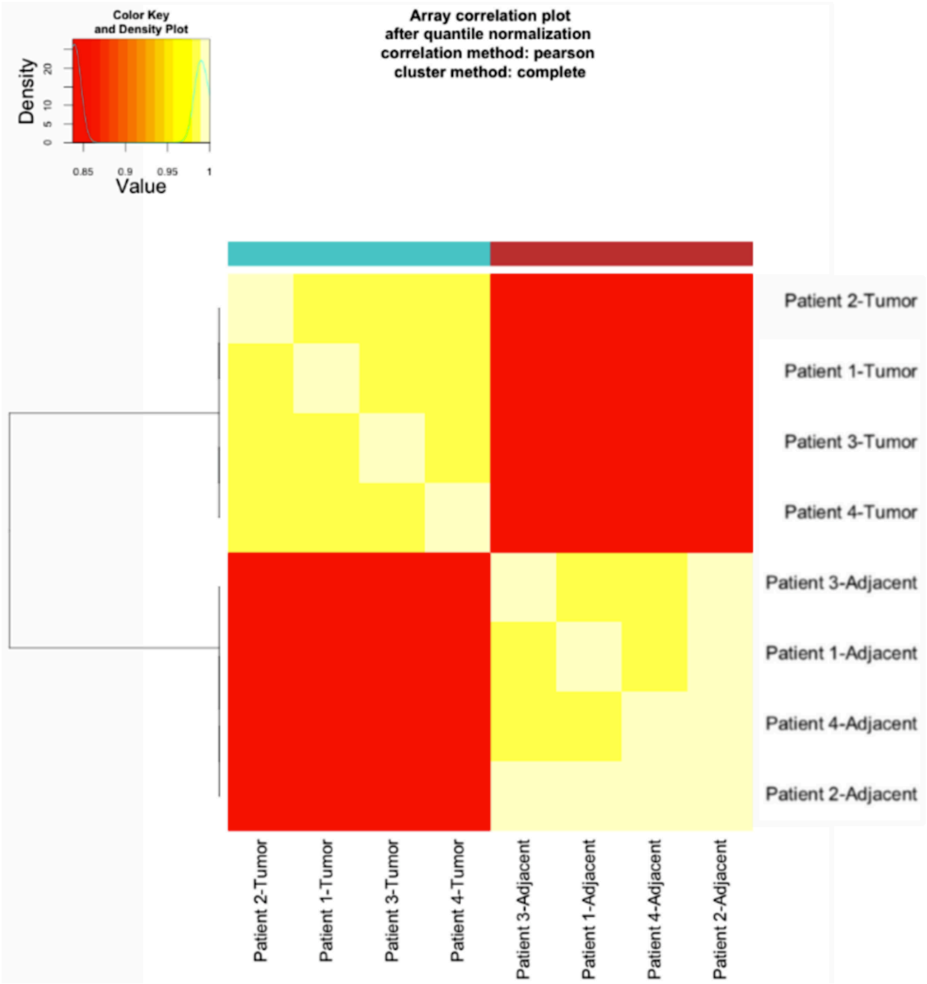

Supplement: Additional file 1: — User guide. [file 12864_2015_1689_MOESM1_ESM.pdf]
