# Supplementary material for: A user-friendly workflow for analysis of Illumina gene expression bead array data available at the arrayanalysis.org portal
Source: BMC Genomics. 2015 Jun 30;16(1):482. doi: 10.1186/s12864-015-1689-8 (PMC4486126; doi:10.1186/s12864-015-1689-8)
Supplement: Additional file 2: — Tutorial demonstrating analysis of a publicly available example dataset from ArrayExpress. [file 12864_2015_1689_MOESM2_ESM.pdf]

Tutorial Illumina Analysis ArrayAnalysis.org

This tutorial serves as a stepwise illustration how to analyze Illumina bead chip gene expression data using the ArrayAnalysis.org web portal. It is meant as an instruction how to apply the workflow to a real data set. More information on all of the options available and on interpreting the module's outputs is provided in the separately provided user guides.

Data set

In order to apply the workflow to example data, you will first download a data set from the ArrayExpress repository at EBI. We will use the data set with code E-MTAB-1064, which you can find at https://www.ebi.ac.uk/arrayexpress/experiments/E-MTAB-1064/.

This data set has been generated by Kolář *et al.** They compared the expression profiles of human fibroblasts that had been co-cultured with human keratinocytes, or transformed epithelial cells – either HaCat or FaDu) to those of fibroblasts cultured alone.

You will need the raw (unprocessed) data in order to explore the functionality of our workflow. This raw data you can find at the bottom of the page, called E-MTAB-1064.raw.1.zip. Download this file to your local computer and extract the content (the file `InterleukinFibroblast_nonorm_nobkgd.txt`) to a destination folder of your choice.

Running the Illumina quality control (QC) and pre-processing module

Open the Arrayanalysis.org web portal and select the **Illumina QC and pre-processing module** from the left menu.

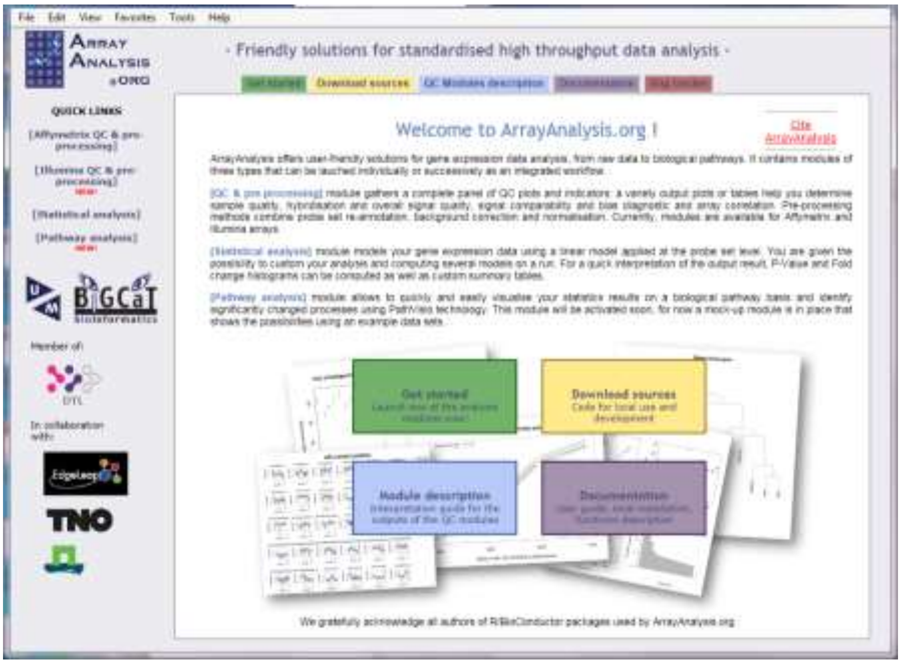

Alternatively, you can click the *Get started* option, and click the module from the list presented, as shown below.

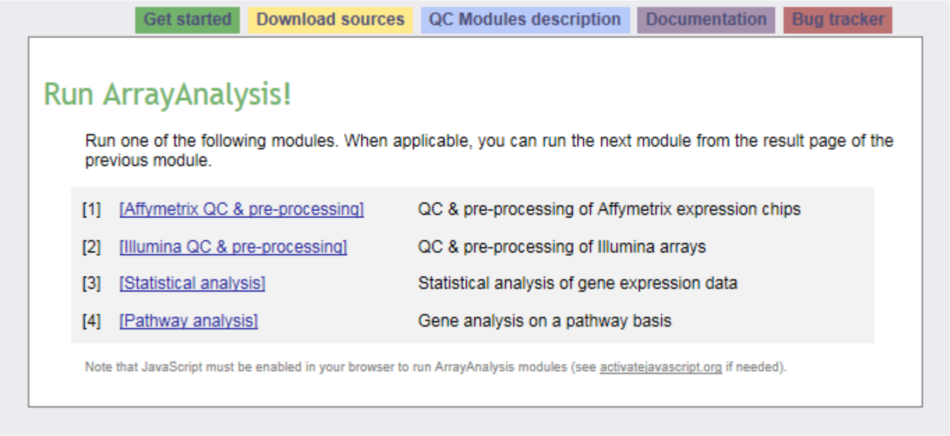

After selecting the module, the opening form shows.

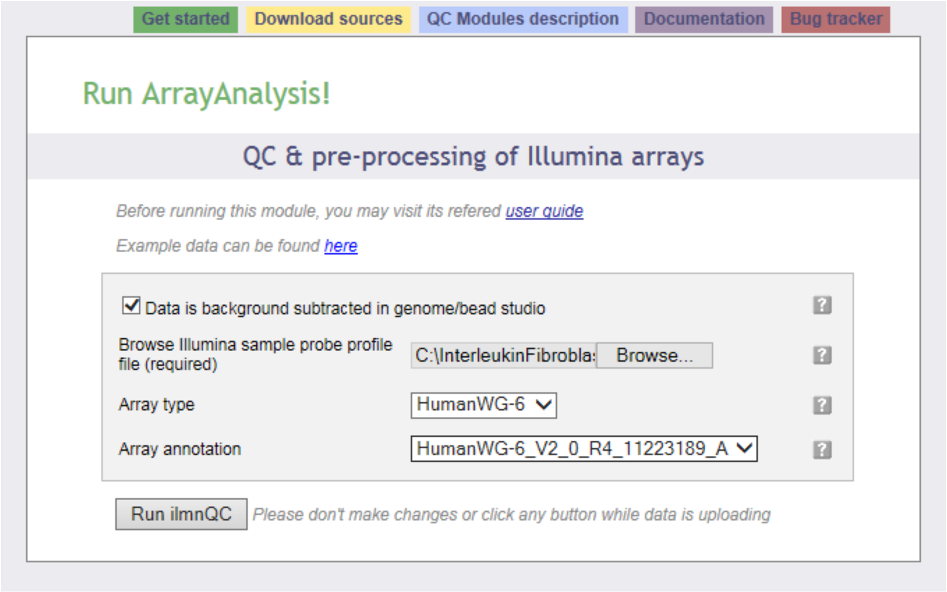

This form allows you to upload the raw data set, and specify which type of bead chip it concerns. In this case tick the *Data is background subtracted* checkbox (we have no control probes file for this data set). Then upload the file that you have extracted from the zip archive before (`InterleukinFibroblast_nonorm_nobkgd.txt`). Thereafter select HumanWG-6 as chip type and V2 as the annotation (in ArrayExpress the chip type for this experiment is given as Illumina Human-6 v2 Expression BeadChip). When done, click the *Run ilmnQC* button. This launches the second form of the module.

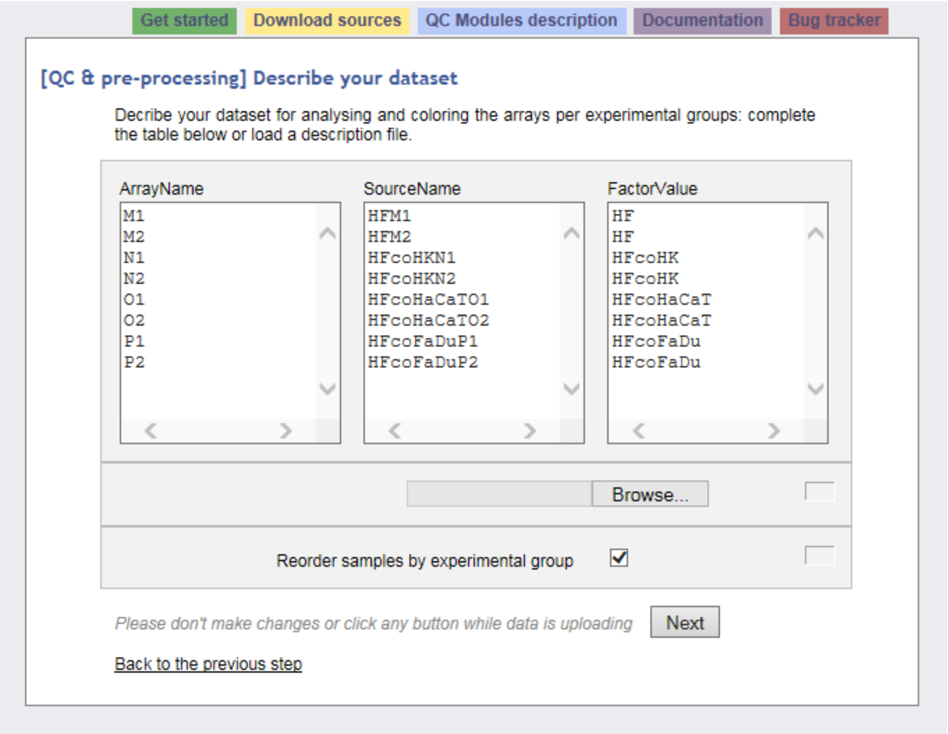

In this form you will find the ArrayName column being autocompleted with the names of the samples as given in the dataset that you have uploaded, in this case M1, …, P2. By completing the SourceName column you can instruct the system how to name the arrays in the plots and results files (you may want to use more informative names than the ones given in the uploaded data set). Finally, by setting the FactorValue column, you can specify which sample belongs to which experimental group. This information is used to color code samples in plots and, if desired, to pass on to the statistics module later.

You can provide this information either by completing it on the web form as shown in the image above, or by uploading a text file with the same information. For your convenience you can copy paste the information from the table below.

ArrayName	SourceName	FactorValue
M1	HFM1	HF
M2	HFM2	HF
N1	HFcoHKN1	HFcoHK
N2	HFcoHKN2	HFcoHK
O1	HFcoHaCaTO1	HFcoHaCaT
O2	HFcoHaCaTO2	HFcoHaCaT
P1	HFcoFaDuP1	HFcoFaDu
P2	HFcoFaDuP2	HFcoFaDu

This information has been obtained from the study description given for this data set in ArrayExpress, in the *Sample and data relationship* file E-MTAB-1064.sdrf.txt, provided right above the raw data. The information in the first column of that file gives the array names

that can be matched with the ArrayName column in the web from. The FactorValue information was obtained from the *Characteristics[GrowthCondition]* column in the annotation file. The SourceName was created by concatenating the FactorValue and the ArrayName, to provide recognizable and unique names for each sample.

Clicking *Next* opens the third and final form that needs to be completed.

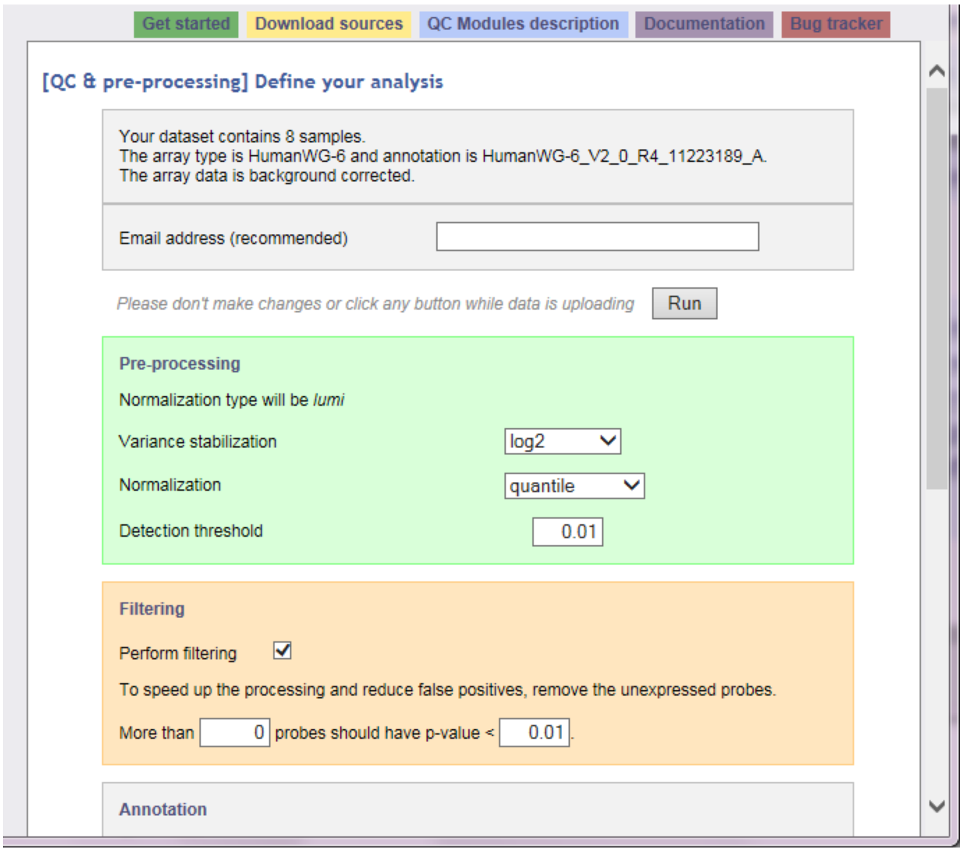

At the top of this form, the system reports some characteristics of your uploaded data set, for example detecting that there are eight samples in the data file. On this page, you can choose several settings that are further documented in the user guide. You can enter your email address if you wish, or just leave it blank. When entering an email address, you will receive a message when the run has finished, otherwise you have to keep the browser open till the run finishes. For now, we leave all other settings to their defaults and click *Run*.

This launches the module. Upon completion, the following screen is shown.

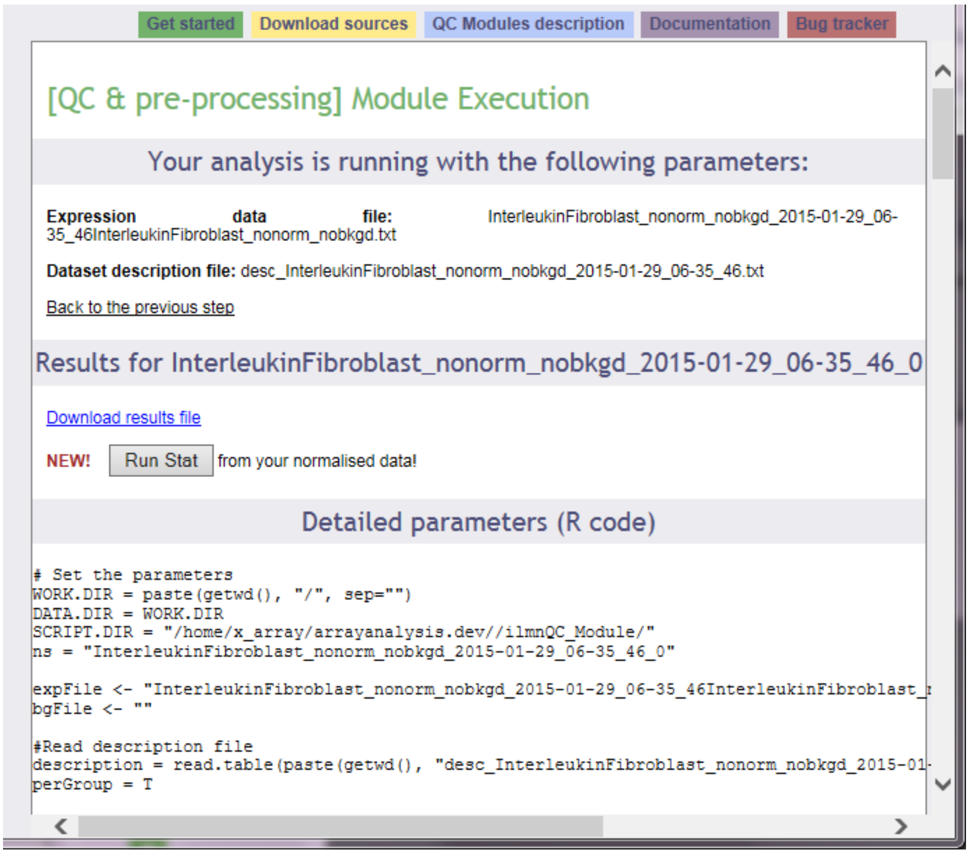

The *Download results file* link, downloads a zip archive with a number of diagnostic QC plots and normalized data tables, as described in more detail in the user guide.

When you are interested in further processing this data set using other ArrayAnalysis modules, click the *Run Stat* button, which sends the data to the Statistics module and opens it.

Running the Statistics module

After clicking the *Run Stat* button from the output screen of the Illumina QC and pre-processing module, the Statistics module opens showing its first form with the data already prefilled based on the information provided earlier, as shown below.

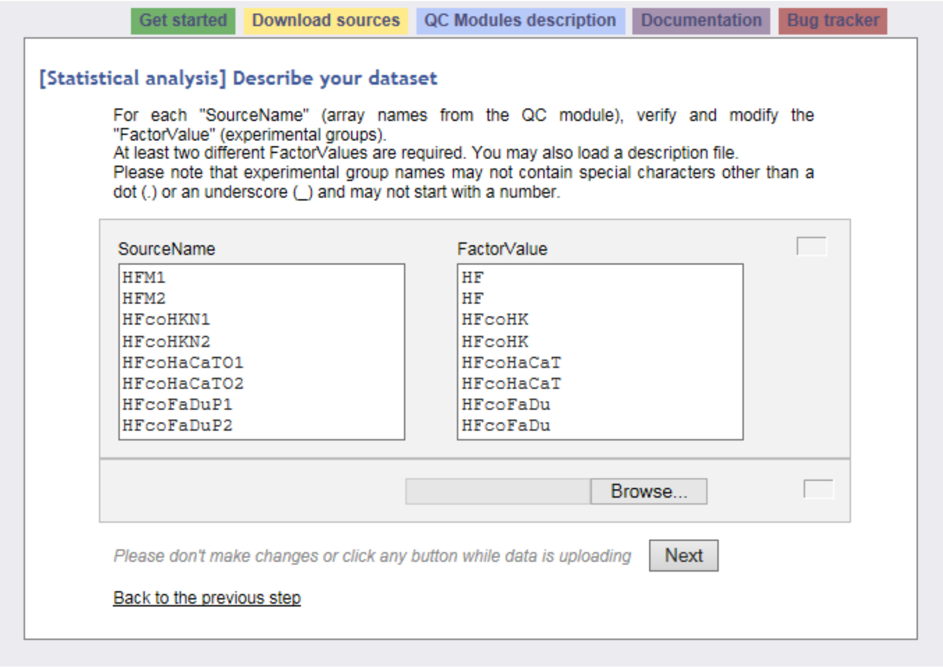

You may change names and groups, but now (as generally) we leave them as they are, and click *Next* to get to the following form.

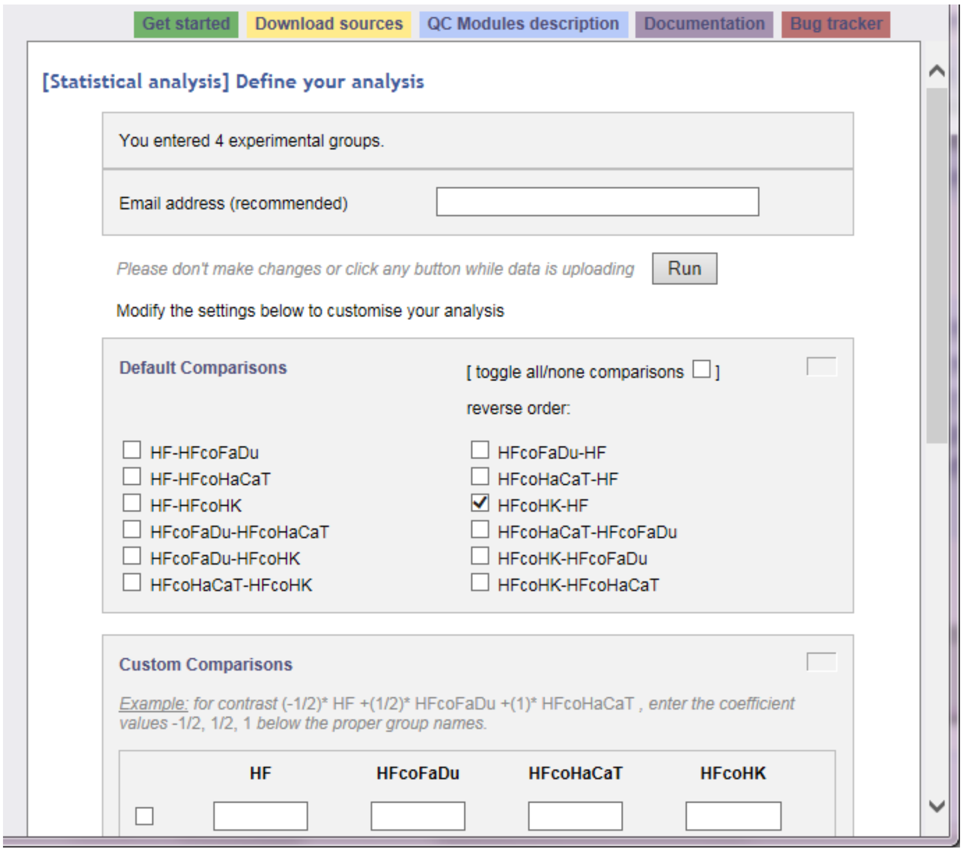

At this form, you can specify several options, as further described in the user guide. For now, enter your email address if you wish to, and only select the HFcoHK-HF comparison as an illustrative example. This compares the fibroblasts co-cultured with keratinocytes to the fibroblasts cultured alone. Just leave all others settings as they are and click Run.

Upon completion the next screen is shown.

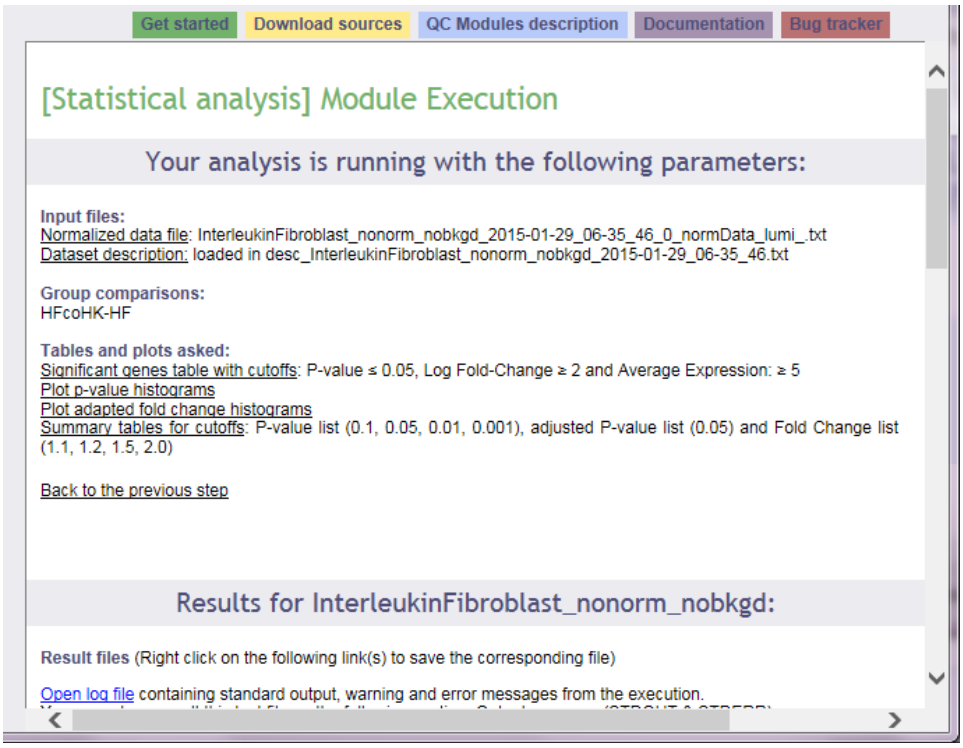

It reports back the settings chosen, and scrolling down it shows the results.

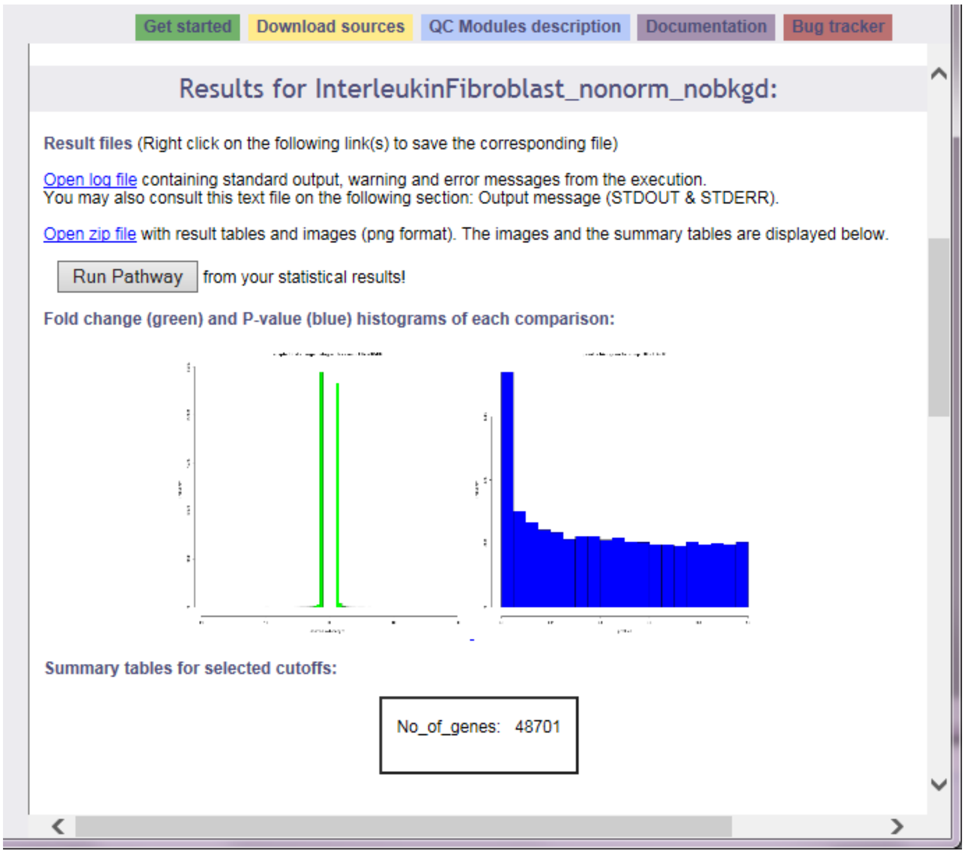

The *Open zip file* link opens an archive containing histogram images, summary tables, and statistical result tables (with fold changes and p values *et cetera*). Also it shows histograms of the fold changes and p values on screen, which are clickable to enlarge. Scrolling down further shows a summary of the number of genes meeting p value and fold change cut-offs as specified before by the user (which we had left to the defaults now).

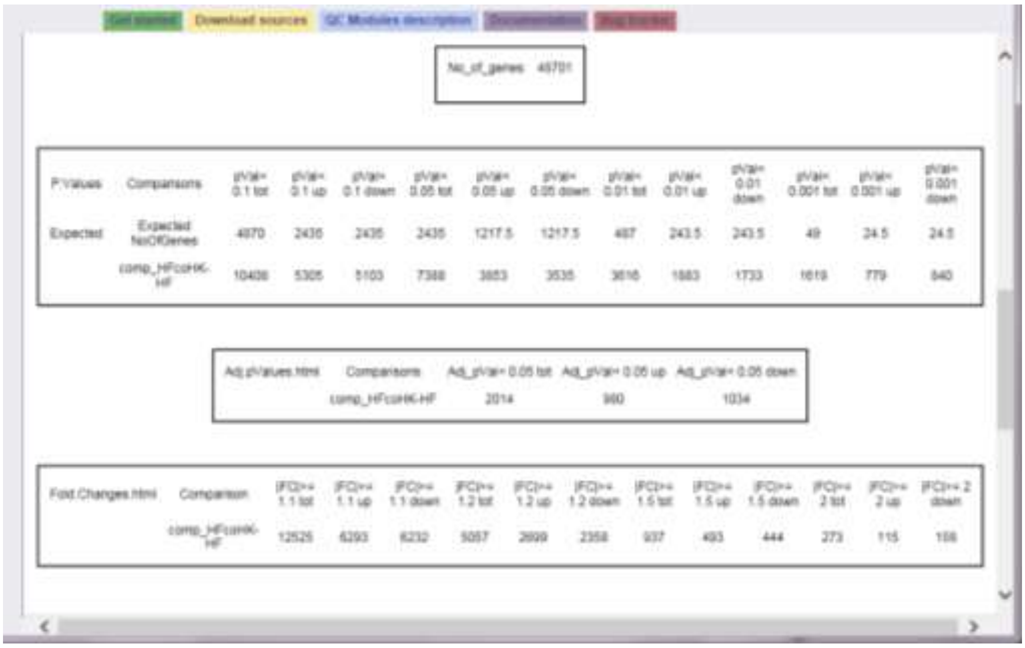

No_of_genes	45701

P.Values	Comparisons	pVal<0.1 tot	pVal<0.1 up	pVal<0.1 down	pVal<0.05 tot	pVal<0.05 up	pVal<0.05 down	pVal<0.01 tot	pVal<0.01 up	pVal<0.01 down	pVal<0.001 tot	pVal<0.001 up	pVal<0.001 down
Expected	Expected NoOfGenes	4870	2435	2435	2435	1217.5	1217.5	487	243.5	243.5	49	24.5	24.5
	comp_HFcoHK-HF	10408	5305	5103	7388	3853	3535	3616	1883	1733	1619	779	840

Adj.pValues.html	Comparisons	Adj_pVal< 0.05 tot	Adj_pVal< 0.05 up	Adj_pVal< 0.05 down
	comp_HFcoHK-HF	2014	980	1034

Fold.Changes.html	Comparison	|FC|>= 1.1 tot	|FC|>= 1.1 up	|FC|>= 1.1 down	|FC|>= 1.2 tot	|FC|>= 1.2 up	|FC|>= 1.2 down	|FC|>= 1.5 tot	|FC|>= 1.5 up	|FC|>= 1.5 down	|FC|>= 2 tot	|FC|>= 2 up	|FC|>= 2 down
	comp_HFcoHK-HF	12525	6293	6232	5057	2699	2358	937	493	444	273	115	158

All results are explained further in the documentation of the Statistics module that can be found at the web portal.

If you wish you can continue performing pathway analysis based on these statistical results, by clicking the *Run Pathway* button, directly above the on-screen histograms.

Running the Pathway module

After clicking the *Run Pathway* button from the Statistics module, the first screen of the Pathway module opens, giving the option to select a statistical comparison of choice. For this statistical comparison, pathway analysis and visualization using programmatic calls to the PathVisio software are performed.

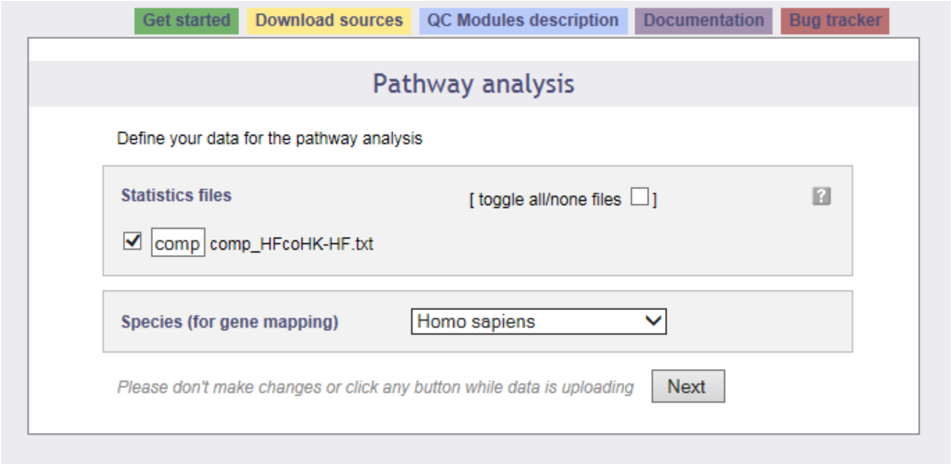

In this case we had only performed on comparison (HFcoHK-HF) which you will find indicated. Check this comparison and set the species to Homo Sapiens. Then click *Next* to open the next form.

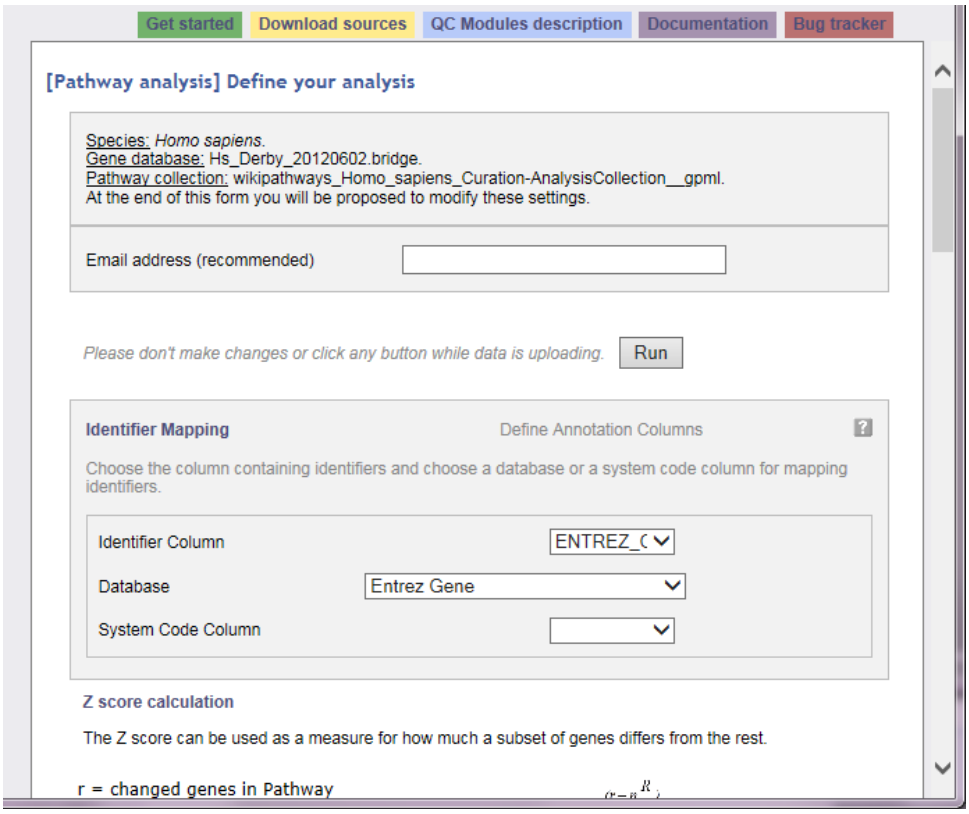

At this form we again leave most settings to the defaults. You can enter your email address if desired. Furthermore we need to specify some information about the identifier we want to use to label the genes in the data set and map the data to the pathways. Let's choose the column `ENTREZ_GENE_ID` (name not fully visible in the screenshot) to work with. Accordingly, select Entrez Gene as the database to use for mapping the identifiers in the column. Then click *Run* to launch the Pathway module.

Upon completion a result screen opens.

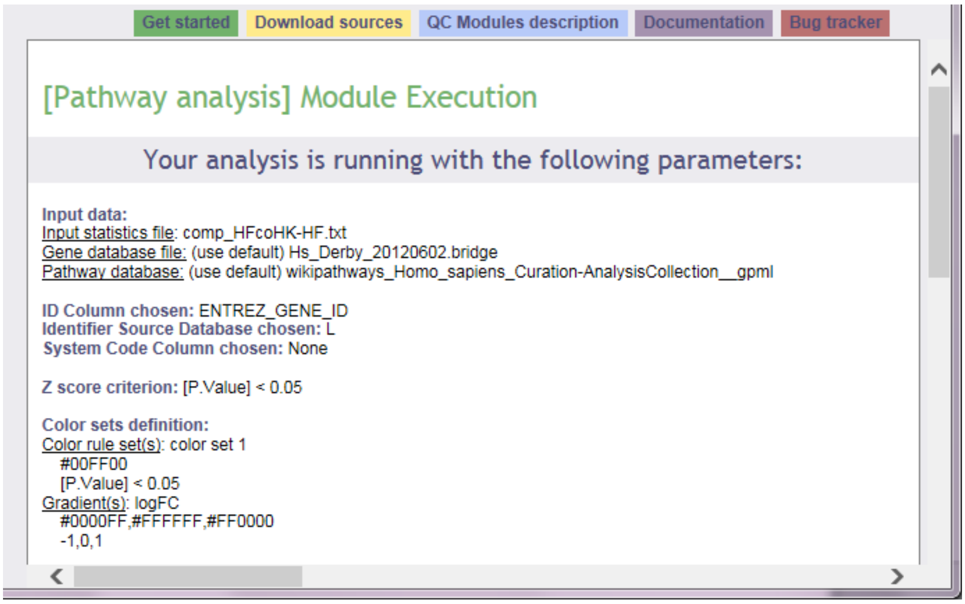

This reports user settings and, when scrolling down, shows the results.

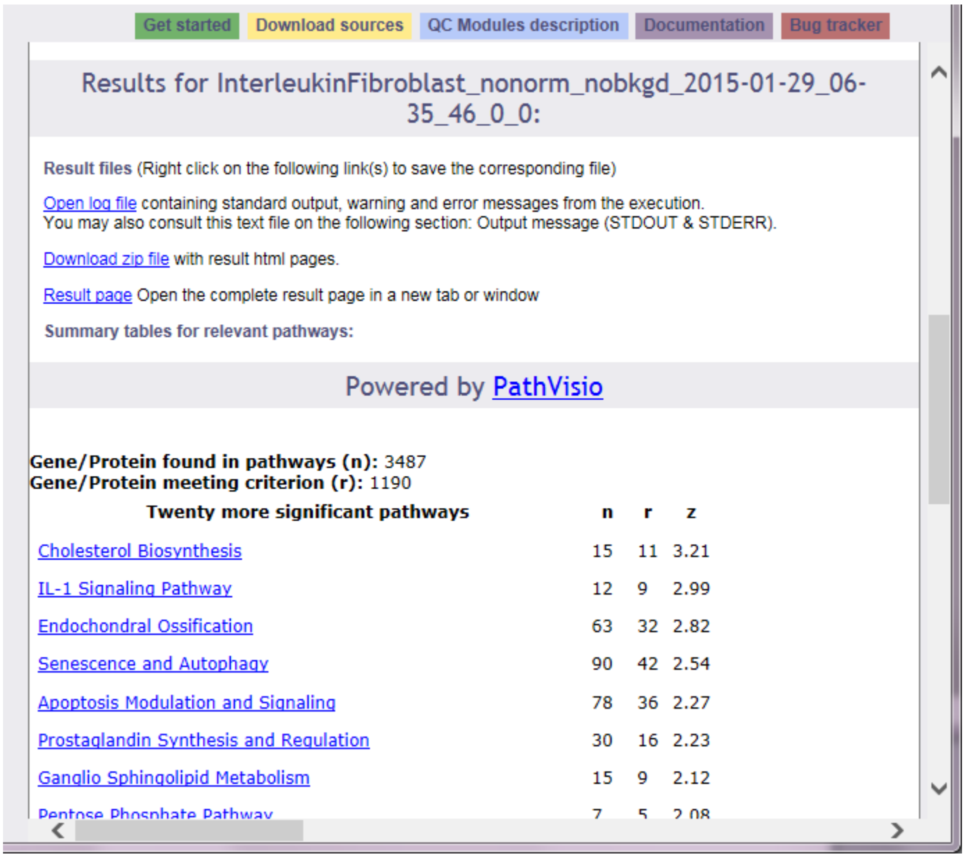

The *Results page* link will lead to a separate page, interactively showing the results, as described below. The *Download zip file* provides the same page for download to the local computer. Thereafter a list of top changed pathways are shown, in a format similar to and described below for the separate results page. Let's turn to that page now.

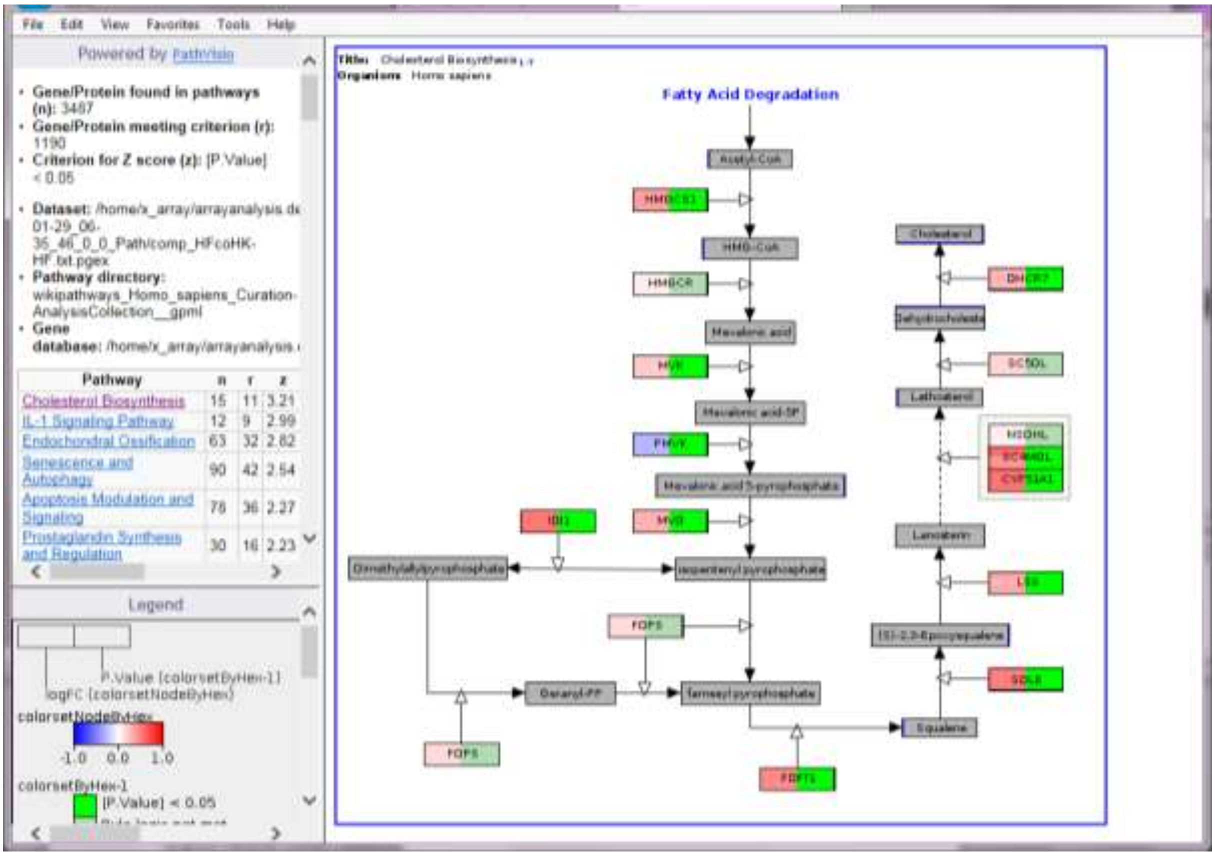

The results page that opens in a separate tab has three panels. Top left, the PathVisio settings used are reported. Also a ranked list of pathways, from most overrepresented to most underrepresented is given. For each pathway, the number of genes/metabolites/proteins it contains (n), the number thereof that meet the criteria specified (r), and the z score for overrepresentation are given. When clicking on one of the pathway names, its pathway diagram, showing the data according to the visualization specified, is opened in the right panel. Note that we had left criteria for pathway statistics as well as visualization options to their defaults now. The screenshot shows the results page with the Cholesterol Biosynthesis pathway opened. The lower left panel shows the legend for the pathway visualization.

Note: you can also enter the workflow from the Statistics or Pathway module, by uploading the table of normalized or statistically analyzed data, respectively.

Note: as the modules remain to be developed, details may be different from those given in (the screenshots in) this tutorial.

Herewith we complete this tutorial describing the analysis of an example Illumina data set in ArrayAnalysis.org.
